# A long diagnostic delay in patients with Hereditary Haemorrhagic Telangiectasia: a questionnaire-based retrospective study

**DOI:** 10.1186/1750-1172-7-33

**Published:** 2012-06-07

**Authors:** Paola Pierucci, Gennaro M Lenato, Patrizia Suppressa, Patrizia Lastella, Vincenzo Triggiani, Raffaella Valerio, Mario Comelli, Daniela Salvante, Alessandro Stella, Nicoletta Resta, Giancarlo Logroscino, Francesco Resta, Carlo Sabbà

**Affiliations:** 1 Geriatric Unit and Rare Disease Center, University Hospital of Bari, Bari, Italy; 2 IRCCS Foundation S. Maugeri, Pavia, Italy; 3 Department of Emergency and Organ Transplantation, “A. Moro” University of Bari, Bari, Italy; 4 Apulia Regional Health Agency, Bari, Italy; 5 Medical Genetics Unit, University Hospital of Bari, Bari, Italy; 6 Department of Neurologic and Psychiatric Sciences, University “A. Moro” of Bari, Bari, Italy; 7 Interdisciplinary Department of Medicine, “A. Moro” University of Bari, Bari, Italy

**Keywords:** Hereditary haemorrhagic telangiectasia, Rendu-Osler-Weber disorder, Vascular malformations, Diagnostic delay, Rare disease

## Abstract

**Background:**

The difficulty in establishing a timely correct diagnosis is a relevant matter of concern for several rare diseases. Many rare-disease-affected patients suffer from considerable diagnostic delay, mainly due to their poor knowledge among healthcare professionals, insufficient disease awareness among patients’ families, and lack of promptly available diagnostic tools. Hereditary Haemorrhagic Telangiectasia (HHT) is an autosomal-dominantly inherited vascular dysplasia, affecting 1:5,000-10,000 patients. HHT is characterized by high variability of clinical manifestations, which show remarkable overlapping with several common diseases.

**Aim:**

To perform a detailed analysis concerning the diagnostic time lag occurring in patients with HHT, defined as the time period spanning from the first clinical manifestation to the attainment of a definite, correct diagnosis.

**Methods:**

A questionnaire was administered to the HHT patients previously recruited from 2000 and 2009. Clinical onset, first referral to a physician for disease manifestations, and first correct diagnosis of definite HHT were collected. Eventual misdiagnosis at first referral and serious complications occurring throughout the time elapsing between disease onset and definite diagnosis were also addressed.

**Results:**

In the 233 respondents, the clinical onset of disease occurred at an age of 14.1 yrs, while the age of first referral and the age of first definite diagnosis of HHT were 29.2 yrs and 40.1 yrs, respectively. Only 88/233 patients received a correct diagnosis at first counseling. Thus, the diagnostic time lag, represented by the time elapsing from disease onset and first definite diagnosis of HHT, proved to be 25.7 yrs. Twenty-two patients suffered from severe complications during this time interval. The diagnostic delay was significantly longer (p < 0.001) in index patients (first patients who attained definite HHT diagnosis in a given family) than in non-index patients (relative of index patients). The diagnostic time lag was also significantly associated with education grade (p < 0.001).

**Conclusions:**

Our data report for the first time a systematic inquiry of diagnostic delay in HHT showing that patients receive a definite diagnosis only after nearly three decades from disease onset. Concerted efforts are still to be made to increase awareness of this disease among both families and physicians.

## Background

Hereditary Haemorrhagic Telangiectasia (HHT), also known as Rendu-Osler-Weber Syndrome, is a rare dominantly-inherited disorder with an estimated prevalence of 1:5,000-10,000, characterized by the presence of epistaxis, mucocutaneous telangiectases, and visceral arterio-venous malformations (AVMs) in the liver, lung, brain and GI tract [1,2]. Age-related penetrance and variable phenotypic expression are commonly found in HHT patients. Thus far, mutations in two genes have been found to be responsible for HHT, namely *ENG* gene for HHT1 and *ACVRL1* gene for HHT2 [3,4].

Several studies have reported that this disease is usually evidenced by spontaneous episodes of epistaxis in childhood or adolescence; however, as epistaxis is a common symptom in the general population, especially during childhood, and is shared with several other diseases, it might easily go unnoticed, rather than recognized as an HHT-related manifestation [5-7]. In many cases, the correct correlation between the first HHT-related manifestations and a definite diagnosis of HHT is not established until other disease features become clinically overt in adulthood, including vascular complications potentially occurring during this time lag. As a consequence, underdiagnosis of HHT patients is a widespread phenomenon; in fact, current figures indicate that up to 90 % of HHT patients in United States still lack a definite diagnosis and proper surveillance [7]. This delay is most likely due to its rarity and to the lack of knowledge concerning the disease on the part of physicians. Hence, the delay in proper diagnosis of HHT merits a more profound examination.

Several studies have explored the length of time required for diagnosis of rare diseases [8-12]. Indeed, the long pathway, often necessary to find the correct diagnosis or treatment, typically determines considerable discomfort on the part of the patients involved with rare diseases, such as HHT, and their families. To our knowledge, there are no previous studies focusing on HHT diagnosis in particular. Therefore, the aim of this study was to investigate the length of time between symptom onset and first definite diagnosis in an HHT patient population.

## Methods

### Patients

A questionnaire was submitted to the HHT patients referring to our HHT Interdepartmental Centre at the University of Bari from 2000 to 2009. The patients were recruited in alphabetic order from our patient database with no selection-based criteria and were considered suitable for the study if they had a definite HHT diagnosis confirmed by either positive genetic testing after identification of the familial causative mutation [13], or presence of at least 3 of 4 Curaçao criteria for HHT clinical diagnosis (epistaxis, mucocutaneous telangiectases, familiarity, AVMs in internal organs [14]) when mutational screening had been inconclusive or still ongoing. All recruited patients had previously attended our Centre and had been subjected to our complete instrumental screening protocol for HHT management as well as mutational screening of *ENG* and *ACVRL1* genes [15]. Henceforth, patients carrying a mutation in *ENG* gene will be referred to as HHT1, patients carrying a mutation in *ACVRL1* gene as HHT2, while patients with unidentified mutation as HHT?. For each patient enrolled, presence of epistaxis, mucocutaneous telangiectases, pulmonary arterio-venous malformations (PAVMs), cerebral arterio-venous malformations (CAVMs), hepatic arterio-venous malformations (HAVMs), GI bleeding, and occurrence of HHT-related complications was retrieved from patient clinical charts collected during hospitalization in our Centre at time of instrumental screening. This study was approved by the ethical committee of the Bari University Hospital.

### Questionnaire

Questionnaires were proposed and administered by means of a telephone interview by one medical doctor with at least a 5-yr training in HHT (P. P). As a first step, patients were asked to provide their explicit consent to participate to the study and only patients who gave their consent were enrolled in the subsequent interview. The questionnaire conceived by our study group was structured with questions aimed to emphasize the exact timing steps required by the diagnostic process for HHT.

The first part of the questionnaire dealt with demographics, familial presence of confirmed HHT, in addition to age of onset and type of first clinical manifestation of the disease. Educational background was classified as follows: a low educational grade (0–7 years), intermediate educational grade (8–12 years), and high educational grade (high school diploma or university degree, >12 education years). Patients were split into two subgroups: “index cases” and “non-index cases”. In the “index cases” group, we included those patients who were the first to receive a definite HHT diagnosis within their family, while in the “non-index cases” group, we included those patients who were definitely diagnosed with HHT as the result of a previous HHT diagnosis for another known family relative. Since our HHT Centre currently recommends genetic testing to the relatives of the index cases whose causative mutation has been identified, also to asymptomatic and pauci-symptomatic individuals, we further performed analysis of non-index cases according to how they received the definite diagnosis: “clinical diagnosis” for those who received the 1^st^ definite diagnosis if positive to at least 3 clinical Curaçao criteria, “genetic diagnosis” for those who resulted positive to genetic testing of the familial mutation, albeit not matching clinical Curaçao criteria for certain diagnosis.

The second part contained questions concerning the first medical referral for HHT-related manifestations with regard to date, place (emergency room, hospital, ambulatory, HHT centre) and specialty of the physician who was consulted for diagnosis. Distinction was made between a general practitioner/family physician and specialist, and also between different specialties (Internal Medicine, Haematology, ENT, Radiology, Gastroenterology). If HHT was not recognized upon first referral, data were also collected regarding the referral in which a definite diagnosis of HHT was made for the first time.

### Statistical analysis

The referral time lag (RTL) was assessed as the difference between the age of the patient’s first referral to a physician due to HHT-related manifestations and the age of HHT onset, whereas the diagnosis time lag (DTL) was defined as the difference between the age of first definite HHT diagnosis and the age of HHT onset. Furthermore, to gain insight into a possible beneficial effect exerted on diagnosis of non-index cases, by a definite diagnosis previously formulated to the index case within the family, we also reported the time lag elapsing between the date of definite diagnosis of the index cases and the date of definite diagnosis of each non-index cases of the same family, defined as “time lag from index diagnosis” (LFID). The statistical significance of the reported data was assessed by univariate analysis (Kruskal-Wallis test or Mann–Whitney test, when appropriate). To better identify underlying factors, analysis of DTL was further performed by multivariate regression analysis. For this purpose, DTL was transformed in a two-class variable (DTL ≤ 15 yrs and DTL > 15 yrs): a logistic model was then set up with DTL being considered as the outcome categorical variable, while age at interview (age ≤ 50 yrs and age > 50 yrs), sex, index/non-index status, educational grade, affected gene, were considered as independent categorical variables. The computations were performed by the statistical program R [16]. A p-value <0.05 was considered significant.

## Results

### Demographics

A total of 269 consecutive HHT patients were subjected to telephone interviews, 36 of whom were excluded from subsequent analysis due to either refusal to participate or incomplete data collection. Therefore, 233 patients were enrolled in the present study (119 M:114 F). The mean age at the time of interview of all 233 enrolled patients was 51.1 years, as the mean age for male vs female patients was 52.4 yrs and 49.7 yrs, respectively. The group of “index cases” was composed of 112 patients and the group of “non-index cases” of 121 patients. A causative mutation had been identified in 213 patients, 81/233 HHT1 (34.8 %) and 132/233 HHT2 (56.5 %), while 20/233 patients (9.4 %) had no mutation identified. Out of the 233 patients, 53 had a low educational level, 69 an intermediate level, and 111 a high level. Demographic and clinical data of the 233 patients are shown in Table 1.

**Table 1:** Demographic data and clinical features of the 233 patients, according to Sex, Education Level, Affected Gene, and Index/Non-Index status

**DEMOGRAPHIC AND CLINICAL DATA**				
**SEX**				
	**Total**	**Males**	**Females**	
**Nr (%)**	**233**	**119 (49%)**	**114 (51%)**	
**Age at Interview, Mean ± SD (years)**	**51.1 ± 16.9**	**52.4 ± 17.2**	**49.7 ± 16.6**	**p = ns**
**[Range]**	**[10;84]**	**[11;81]**	**[10;84]**	
**EDUCATION LEVEL**				
	**Low**	**Interm**	**High**	
**Nr (%)**	**53 (23%)**	**69 (30%)**	**111 (47%)**	
**Age at Interview, Mean ± SD (years)**	**62.5 ± 16.9**	**49.9 ± 16.2**	**46.4 ± 14.9**	**P < 0.0001**
**[Range]**	**[10;84]**	**[15;78]**	**[16;75]**	
**AFFECTED GENE**				
	**HHT1**	**HHT2**	**HHT?**	
**Nr (%)**	**81 (35%)**	**132 (56%)**	**20 (9%)**	
**Age at Interview, Mean ± SD (years)**	**46.6 ± 16.9**	**52.4 ± 16.8**	**60.1 ± 13.3**	**P < 0.003**
**[Range]**	**[11;79]**	**[10;84]**	**[36;80]**	
**INDEX/NON-INDEX STATUS**				
	**Index**	**Non-Index**		
**Nr (%)**	**112 (48.1%)**	**121 (51.9%)**		
**Age at Interview, Mean ± SD (years)**	**57.4 ± 14.1**	**45.2 ± 17.3]**		**P < 0.0001**
**[Range]**	**[15;81]**	**[10;84]**		
**CLINICAL DATA**	**Nr (%)**			
**Epistaxis**	**231 (99.1%)**			
**Mucocutaneous Telangiectases**	**233 (100%)**			
**PAVM**	**117 (50.2%)**			
**CAVMs**	**19 (8.1%)**			
**HAVMs**	**182 (78.1%)**			
**GI bleeding**	**62 (26.6%)**			

### First manifestation of HHT disease

Among the 233 patients enrolled, 231 had manifested overt symptoms related to HHT by the time of the interview. The mean age at onset was 14.1 ± 11.2 years [range 3–60]. Data regarding clinical onset are shown in Table 2. Two adult patients who were first-degree relatives of definitely affected individuals reported no subjective manifestations of HHT disease until the date of interview. For these two patients, diagnosis of HHT was obtained by genetic testing for the previously-identified familial mutation. A subsequent medical evaluation noted a few mucocutaneous telangiectases, previously unrecognized by the patients, while instrumental screening evidenced silent HAVMs in both subjects and PAVMs in one of them. An additional three patients (two of which in paediatric age) had no subjective manifestations at the time of diagnosis (which was performed by genetic testing), although they suffered from spontaneous epistaxis before the time of the interview.

**Table 2:** First manifestation of HHT disease in the 233 patients, according to Sex, Education Level, Affected Gene, and Index/Non-Index status

**ONSET OF HHT**				
**SEX**				
	**Total**	**Males**	**Females**	
**Onset Age, Mean ± SD (years)**	**14.1 ± 11.2**	**14.6 ± 12.6**	**13.6 ± 9.7**	**p = ns**
**[Range]**	**[3;60]**	**[3;60]**	**[3;50]**	
**EDUCATION LEVEL**				
	**Low**	**Interm**	**High**	
**Onset Age, Mean ± SD (years)**	**17.2 ± 13.4**	**12.2 ± 9.8**	**13.8 ± 10.7**	**p = 0.115**
**[Range]**	**[3;51]**	**[3;60]**	**[3;58]**	
**AFFECTED GENE**				
	**HHT1**	**HHT2**	**HHT?**	
**Onset Age, Mean ± SD (years)**	**9.9 ± 8.6**	**16.6 ± 12.0**	**17.4 ± 14.4**	**P < 0.0001**
**[Range]**	**[3;47]**	**[3;58]**	**[4;60]**	
**INDEX/NON-INDEX STATUS**				
	**Index**	**Non-Index**		
**Onset Age, Mean ± SD (years)**	**16.2 ± 13**	**12.1 ± 9.1**		**P < 0.02**
**[Range]**	**[3;60]**	**[3;49]**		
**CLINICAL ONSET**	**Nr (%)**			
**Epistaxis alone**	**202 (86.7%)**			
**Epistaxis and Mucocut. Tel.**	**15 (6.4%)**			
**Epistaxis and GI bleeding**	**2 (0.9%)**			
**Epist., Mucocut. Tel, and GI bleeding**	**1 (0.5%)**			
**Mucocut Tel. (bleeding + non-bleeding)**	**6 (2.6%)**			
**GI bleeding**	**2 (0.9%)**			
**PAVM-related Accidents**	**2 (0.9%)**			
**CAVM-related Accidents**	**1 (0.5%)**			
**Asymptomatic (only Genetic testing)**	**2 (0.9%)**			

Epistaxis represented the onset of HHT disease in 220/233 (94.4%) index cases. Epistaxis alone was reported to be the first manifestation in 202/233 (86.7%) in this group, whereas in 18/233 (7.7%) patients additional manifestations were reported to occur at the same age as epistaxis onset: appearance of mucocutaneous telangiectases was reported by 15 patients, GI bleeding episodes by 2 patients, while one patient reported both visible mucocutaneous telangiectases and overt GI bleeding episodes occurring at the same age as the first nosebleeds. A cerebrovascular accident occurred as the onset of disease in 3/233 (1.3%) otherwise asymptomatic individuals: brain abscess secondary to PAVMs in 2 cases, while seizures secondary to large CAVMs occurred in one case. Onset of non-bleeding mucocutaneous telangiectases and appearance of bleeding lip telangiectases occurred in five patients and in one patient, respectively. The overall age at first physician counselling for HHT-related symptoms was 29.2 ± 16.0 years [range 3;75] for all 233 patients, with an RTL of 14.9 ± 14.4 years [range 0;65].

### First definite diagnosis of HHT

Of the 233 patients included in the present study, a definite diagnosis for HHT was received at the age of 40.1 ± 17.2 years [range 3;78]. Only 88/233 (37.8%) patients attained the definite diagnosis of HHT at the first referral. Among the 112 index cases, only 28 (25.0%) received a definite diagnosis of HHT at the first counselling. Regarding the remaining 84 patients, 48 (42.8%) received an incorrect diagnosis and 36 (32.2%) did not receive any diagnosis. Among the 121 non-index cases, 60 (49.8%) received a definite diagnosis of HHT at first counselling, 41 of which were formulated by HHT Centres. In the remaining 61 patients who did not receive a correct diagnosis of HHT upon first referral, an incorrect diagnosis was provided to 36 patients, whereas no diagnosis was formulated for 25 patients. The incorrect diagnoses included: “*fragility of capillary vessels*” in 66 cases, “ *angiomas*” (labial, lingual, hepatic, congenital) in 5 cases, “ *tumoral mass lesions*” in 3 cases, whereas the following wrong diagnosis were each formulated in 1 case: “ *hemoptysis from pulmonary angioma*”, “ *multiple non-syndromic pulmonary arteriovenous fistulas*”, “ *anaemia*”, “ *diffuse angiodysplasia*”, “ *bleeding cerebral abscess*”, “ *nasal polyps*”, “ *congenital disorder*”, “ *sinusitis*”, “ *thrombocytopenia secondary to depression*”, “ *acute cholecystitis*”. Data regarding the 1^st^ definite diagnosis of HHT in the various subgroups are shown in Table 3.

**Table 3:** First definite diagnosis of HHT in the 233 patients, according to Sex, Education Level, Affected Gene, and Index/Non-Index status

**FIRST DEFINITE DIAGNOSIS OF HHT**				
**SEX**				
	**Total**	**Males**	**Females**	
**Age at Diagnosis, Mean ± SD (years)**	**40.1 ± 17.2**	**41.2 ± 17.6**	**38.9 ± 16.8**	**p = ns**
**[Range]**	**[3;78]**	**[7;78]**	**[3;73]**	
**EDUCATION LEVEL**				
	**Low**	**Interm**	**High**	
**Age at Diagnosis, Mean ± SD (years)**	**53.5 ± 17.4**	**37.1 ± 16**	**35.5 ± 14.5**	**P < 0.0001**
**[Range]**	**[3;78]**	**[4;71]**	**[7;73]**	
**AFFECTED GENE**				
	**HHT1**	**HHT2**	**HHT?**	
**Age at Diagnosis, Mean ± SD (years)**	**37.6 ± 16**	**39.9 ± 17.5**	**50.6 ± 17.0**	**P < 0.02**
**[Range]**	**[4;77]**	**[3;78]**	**[20;74]**	
**INDEX/NON-INDEX STATUS**				
	**Index**	**Non-Index**		
**Age at Diagnosis, Mean ± SD (years)**	**45.9 ± 15.3**	**34.6 ± 17.1**		**P < 0.001**
**[Range]**	**[7;78]**	**[3;77]**		

### Diagnostic time lag (DTL)

In the overall group of 233 patients, definite diagnosis of HHT was obtained after a DTL of 25.7 ± 17.4 years from the onset of HHT manifestations, with a wide range both in index and non-index patients (from within one year to 73 years). Regarding the index cases, only 3/112 (2.7%) patients were diagnosed as having definite HHT within the first year after the disease onset, and only 2/112 (1.8%) within the second year. On the other hand, 15/112 (13.3%) index patients had to wait 50 years or longer to receive a definite HHT diagnosis. In the non-index subgroup, only 7/121 (5.8%) patients had a definite HHT diagnosis within the first year after the disease onset and only 3/121 (2.5%) within the second year (with five additional patients with positive genetic testing before HHT onset), while 9/121 (7.4%) patients had to wait longer than 50 years. In the multivariate analysis, DTL was significantly correlated with age at interview (p < 0.0001) and educational level (p < 0.05), and with index/non-index status (p < 0.05) and educational level (p < 0.01) after removal of the age from the model. Conversely, neither did univariate or multivariate analysis show any significant difference in DTL between male and female patients, either before or after removal of the age from the model (Table 4). No significant difference was shown by univariate analysis with respect to affected gene (Table 4). Although the affected gene seemed to be significantly associated to DTL by multivariate analysis (p < 0.01), the association was not significant after removal of the age from the model.

**Table 4:** Diagnosis Time Lag (DTL) in the 233 patients, according to Sex, Education Level, Affected Gene, and Index/Non-Index status

**DIAGNOSIS TIME LAG (DTL)**						
				**Univariate and Multivariate Analysis**		
				**(a)**	**(b)**	**(c)**
**SEX**						
	**Total**	**Males**	**Females**			
**DTL, Mean ± SD (years)** **[Range]**	**25.7 ± 17.4**	**26.4 ± 19.2**	**25.1 ± 15.6**	**p = ns**	**p = ns**	**p = ns**
	**[0;73]**	**[0;73]**	**[0;62]**			
**EDUCATION LEVEL**						
	**Low**	**Interm**	**High**			
**DTL, Mean ± SD (years)** **[Range]**	**36.4 ± 18.0**	**24.5 ± 17.2**	**21.4 ± 15.2**	**P < 0.0001**	**P < 0.05**	**P < 0.01**
	**[0;73]**	**[0;65]**	**[0;64]**			
**AFFECTED GENE**						
	**HHT1**	**HHT2**	**HHT?**			
**DTL, Mean ± SD (years)** **[Range]**	**27.7 ± 16.6**	**23.4 ± 17.3**	**33.1 ± 19.6**	**p = ns**	**P < 0.01**	**p = ns**
	**[0;68]**	**[0;73]**	**[2;61]**			
**INDEX/NON-INDEX STATUS**	**Index**	**Non-Index**				
**DTL, Mean ± SD (years)** **[Range]**	**29.1 ± 18.0**	**22.6 ± 16.7**		**P < 0.01**	**p = ns**	**P < 0.05**
	**[0;73]**	**[0;68]**				

Univariate and multivariate analysis were performed as described in Methods section. (a) Univariate analysis, (b) Multivariate analysis before removal of age, (c) Multivariate analysis after removal of age.

### Effect of Index/non-index status

**Figure 1:**
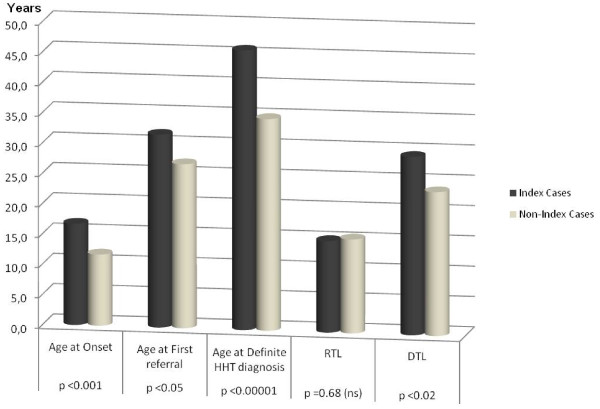
**Timing steps of diagnosis according to Index/Non-Index Status.** RTL = Referral Time Lag; DTL = Diagnosis Time Lag.

The HHT disease arose at the age of 16.2 ± 13 years in index cases vs 12.1 ± 9.1 years in non-index cases (p-value <0.001; Figure 1). The age at 1^st^ referral for HHT-related manifestations was 31.7 ± 15.9 years [range 6;71] vs 26.9 ± 15.8 years [range 3;75] for index cases vs non-index cases (p < 0.05; Figure 1), respectively. Thus, the RTL was 14.9 ± 14 years [range 0;64] vs 14.8 ± 14.7 years [range 0;65] for index cases vs non-index cases, respectively (p = ns). Among the index cases, the first referral for HHT-related features was to a general practitioner in 8/112 patients (7.1%), an HHT centre in 5/112 patients (4.4%), an ENT doctor (62.5%) in 70/112 patients, while 29/112 patients referred to other specialists (25.9%). In the non-index-cases subgroup, the first referral for HHT-related features was to a general practitioner in 8/121 patients (6.7%), to an HHT Centre in 41/121 patients (33.7%), to an ENT doctor in 43/121 patients (35.6%), and to another specialist in 29/121 patients (23.9%). The proportion of patients who referred to an HHT Centre was significantly different (p < 10^-7^) between index and non-index cases. In the index group the definite diagnosis was obtained at age 45.9 ± 15.3 years, compared to an age of 34.6 ± 17.1 years in the non-index cases (p < 0.00001, Figure 1). In particular, the DTL was 29.1 ± 17.8 years in the index cases vs. 22.6 ± 16.7 years in the non-index cases (p-value <0.001 at univariate analysis; Figure 1). Although in the initial step of multivariate analysis the DTL did not seem to be significantly correlated with index/non-index status, removal of age from the model disclosed a statistically significant contribution of index/non-index status to the DTL (p <0.05; Table 4).

When non-index cases were analyzed according to how they received the definite diagnosis, those with genetic diagnosis had a significantly lower DTL than those with clinical diagnosis (14.4 ± 14.8 years [range 0;65] vs. 27 ± 16.2 years [range 0;68], respectively; p < 0.001). Similarly, the RTL of non-index cases with genetic diagnosis was significantly lower (10.9 ± 14.6 years [range 0;65]) than RTL of non-index cases with clinical diagnosis (16.7 ± 14.9 years [range 0;62]; p < 0.05). When considering the non-index cases, the diagnostic delay consistently dropped down from a DTL of 22.6 ± 16.6 years to a value of 6.4 ± 8.4 years if calculated as LFID (i.e. the difference between date of diagnosis of the index case and date of diagnosis of the non-index cases of the same family).

### Effect of educational level

**Figure 2:**
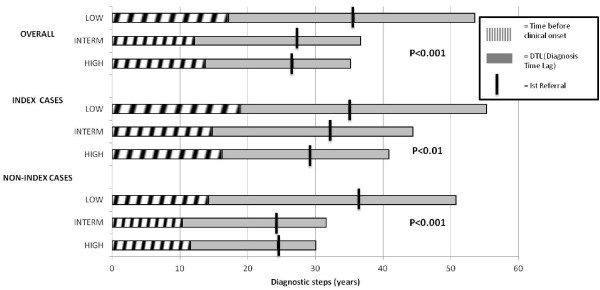
**Referral Time Lag (RTL) and Diagnosis Time Lag (DTL) according to Index/Non-Index Status and Grade of Education.** For each subgroup, the mean value of age of onset, age of 1st referral, and age of 1st definite HHT diagnosis are indicated. In X-axis the time before clinical onset is shown by a white dashed bar. The time comprised between clinical onset and attainment of definite diagnosis of HHT (corresponding to DTL) is shown by a grey full bar. For each subgroup, the mean age of first referral to a physician is labeled by a black vertical rod.

The different educational levels proved to be inversely correlated with the length of DTL; in fact, the diagnostic delay was reported at its highest value (36.4 ± 18.0 yrs) in the patients characterized by a low educational grade, but it was considerably reduced in the patients with intermediate educational level (24.5 ± 17.2 yrs), and it improved even more in those with higher educational levels (21.4 ± 15.2 yrs). As shown in Figure 2, this difference was statistically significant in both index and non-index cases (p < 0.01). Multivariate analysis also indicated a significant effect of educational level on DTL (p < 0.05) before and after removal of age at interview from the model.

### Clinical complications

**Figure 3:**
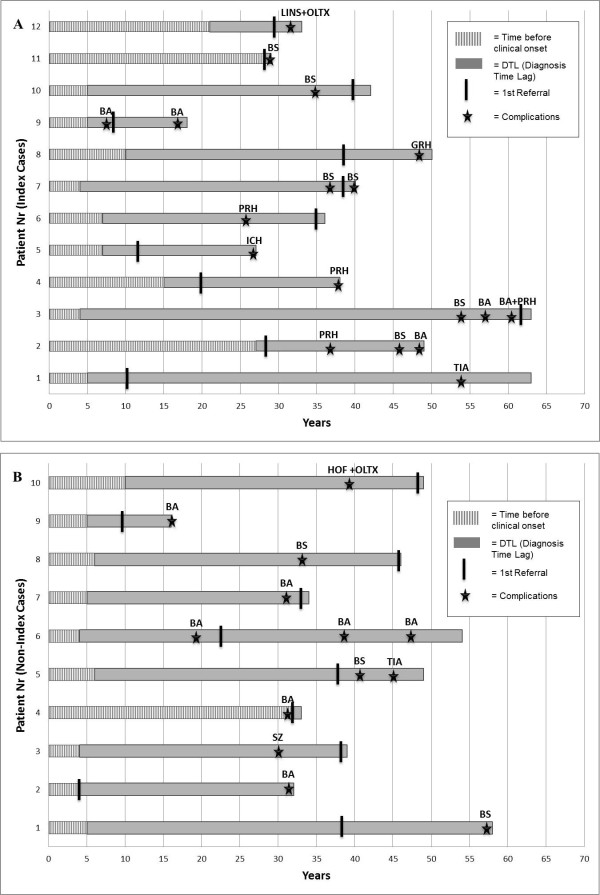
**(Panel A: Index patients; Panel B: Non-index patients). Twenty-two patients suffered from severe complications before attainment of HHT definite diagnosis**. Each horizontal bar represents one given patient - identified by a number on Y-axis - who suffered from AVM-related complications over the time of Diagnosis Time Lag (DTL). In X-axis the time before clinical onset is shown by a white dashed bar. The time comprised between clinical onset and attainment of definite diagnosis of HHT (corresponding to DTL) is shown by a grey full bar. For each patient, the first referral to a physician is labeled by a black vertical rod. Star-shaped markers indicate complication events: BA = Brain Abscess; BS = Brain Stroke; TIA = Transient Ischemic Attack; ICH = Intracranial Haemorrhage; PRH = Pulmonary Haemorrhage; GRH = Haemorrhage from Large Gastrointestinal Colonic AVM; SZ = Seizures; HOF = High-Output Heart Failure; LINS = Liver Insufficiency; OLTx = Orthotopic Liver Transplantation.

A total of 22 patients (12 index and 10 non-index) were affected by severe life-threatening complications secondary to visceral AVMs during the time lag elapsing HHT onset and the correct definite HHT diagnosis (i.e. the DTL), as shown in Figure 3. Among the index cases, 12/112 (10.6%) patients suffered from complications during DTL, with four patients suffering from more than one complication (a total number of 18 episodes). Similarly, in the non-index group, 10/121 (8.3%) patients suffered from unexpected complications over the DTL period, with three patients being affected by more than one complication event, with a total of 13 episodes. Lastly, in three additional patients (two index and one non-index cases), severe sudden complications represented the actual onset of HHT disease (as described in “First manifestation of HHT Disease” paragraph). In twenty of the 22 complication-affected patients, life-threatening accidents were secondary to large cerebral, pulmonary, and colonic AVMs, and would have likely been avoided by appropriate preventive treatment, whereas in the remaining two patients complications were secondary to large hepatic AVMs and thus far required OLTx as only effective therapeutic option.

## Discussion

The astonishing finding arising from this study is the long duration of the time lag occurring between the onset of HHT-related manifestations and the formulation of a definite HHT diagnosis, which resulted to elapse for an average period of almost three decades. Even though several publications have acknowledged that misdiagnosis often occurs in HHT disease [3,17,18], this is the first report of a systematical inquiry of diagnostic time delay (DTL) regarding HHT. The difficulty in obtaining the correct diagnosis proved to represent a dramatic barrier for most HHT patients. This issue represents a general matter of concern for the majority of rare diseases; in fact, this long-lasting search for diagnosis entails numerous referrals to various specialists as well as several instrumental examinations, representing a time-consuming process which is frequently inconclusive since patients often receive incorrect diagnoses. Naturally, this frustrating delay considerably affects the patients’ quality of life, with a subsequent worsening of their clinical status and marked psychological distress, for both patients and their families [12].

Furthermore, in the specific case of HHT, a late diagnosis delays the beginning of proper treatment. The lack of a correct HHT diagnosis prevented the patients from benefiting from safe and effective preventive treatment for visceral AVMs carrying marked clinical significance. In fact, current guidelines for HHT recommend preemptive radiologic survey and treatment for pulmonary AVMs and outline best practice management for AVMs and complications in other organs [19]. In our study, sudden severe complications secondary to visceral AVMs occurred in 22 patients during the DTL. Since these vascular accidents are potentially life-threatening and characterized by considerable disability sequelae, they often result in need for costly neuromotor support and/or pharmacological treatments. Hence, the necessity for a prompt diagnosis of HHT can be also emphasized not only in terms of improved patients’ quality of life (which should itself represent the most relevant aim of health care), but also in terms of economic impact. A rapid diagnosis of HHT would thus permit to reduce the financial burden of such permanent aftermath on families and/or health care agencies [20].

Based on our results of this study, there is still an extensive need to further disseminate knowledge of HHT among the community of physicians as well as among relatives of patients with a definite diagnosis of HHT, thus reducing diagnostic delay and providing optimal management to as-yet undiagnosed HHT patients. As the majority of definite HHT diagnoses were formulated by HHT Centres, the present work stresses the relevance of the role played by these institutions [21] in providing a definite diagnosis of HHT within acceptable time limits. Moreover, our results firmly suggest the existence of several as-yet undiagnosed HHT patients. Special efforts should be made to establish a network involving ENT specialists and family physicians in the activity of HHT Centres, based on the observation that most patients, both index and non-index, initially refer to such medical professionals when subjected to recurrent nosebleeds.

### Reasons underlying the diagnostic delay

**Figure 4:**
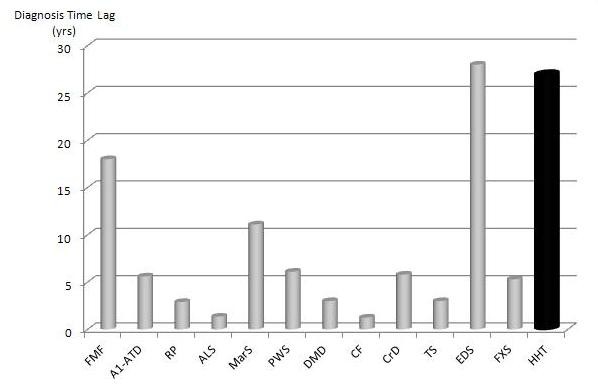
**Diagnostic Time Lag (DTL) in several Rare Diseases.** In Y-axis, the DTL is shown for the following rare diseases: FMF = Familial Mediterranean Fever [9]; A1-ATD = Alfa-1 Antitrypsin Deficiency [10]; RP = Relapsing Polychondritis [11]; ALS = Amyotrophic Lateral Sclerosis [9]; MarS = Marfan Syndrome [12]; PWS = Prader-Willi Syndrome [12]; DMD = Duchenne Muscular Dystrophy [12]; CF = Cystic Fibrosis [12]; CrD = Crohn’s Disease [12]; TS = Tuberous Sclerosis [12]; EDS = Ehlers-Danlos Syndrome [12]; FXS = Fragile-X Syndrome [12]; HHT = Hereditary Haemorrhagic Telangiectasia (this work).

The main reason accounting for such a long diagnostic time lag most likely lies in the insufficient knowledge of HHT disease by the medical community due to its low prevalence, similar to that of many other rare diseases. To gain a better insight into this issue, we decided to compare the diagnostic lag found for HHT to that previously reported for other rare diseases. After completion of this study, a review of current literature for several rare diseases was performed, including the Eurordiscare studies [12]. These studies reported that the arc of time required to reach a correct diagnosis largely varies for the different rare diseases (summarized in Figure 4) [8-12]. When comparing our results to studies focusing on diagnostic delay, the DTL for HHT was one of the longest (25.7 years). Several explanations can be evoked to account for such a long diagnostic delay. (i) Firstly, HHT is a highly heterogeneous disorder lacking a specific clinical hallmark and is characterized by clinical features which overlap with several unrelated common diseases, thus obscuring more specific underlying signs and leading to misdiagnosis [7,22]. In fact**,** it is noteworthy that those diseases with symptoms mainly unspecific and/or quite overlapping with many common diseases such as Familial Mediterranean Fever or Ehlers-Danlos syndrome, proved to be more prone to misdiagnosis and to have the highest DTLs. On the contrary, those rare diseases characterized by specific clinical/instrumental signs, such as the Prader-Willi Syndrome or Tuberous Sclerosis, require a much shorter DTL than HHT (Figure 4). (ii) Secondly, the longer DTL for HHT may also be attributable to the lack of any reliable biochemical test for HHT diagnosis, whereas such laboratory tests are available for other disorders [2]. As reported in Figure 4, diagnostic delay is shorter for those diseases which can currently rely on effective biochemical/histological tests, such as cystic fibrosis, Alpha-1 anti-trypsin deficiency and Crohn’s disease. In spite of the studies of several research groups, no accurate biochemical tests currently exist to diagnose HHT [23-28]. (iii) A third explanation consists in the intrinsic clinical features of HHT itself, which tends to have less severe manifestations and a better clinical evolution than many of the other aforementioned rare diseases. In fact, those diseases with rapid deterioration of psychomotor abilities, such as Amyotrophic Lateral Sclerosis, Cystic Fibrosis, and Duchenne’s Muscular Dystrophy, have a shorter DTL. On the other hand, diseases involving a low neuromotor impairment, such as Familial Mediterranean Fever, or those which remain silent for decades and then precipitate sudden life-threatening events, such as Ehlers-Danlos Syndrome, have a longer DTL. Clinical features of HHT are characteristic of this latter disease group.

### Factors affecting diagnostic delay

Our study suggests that the DTL in HHT is significantly associated with two major factors: index/non-index status and educational level. Our results show that the index cases, who have no known relatives with a definite diagnosis of HHT, undergo an even longer diagnostic delay (29.1 years, represented by 14.9 years of RTL followed by 14.2 years awaiting for the correct diagnosis). Interestingly, we noted that non-index patients were not only properly diagnosed at a younger age than index patients, but also reported a statistically significant earlier clinical onset and earlier age at first referral. These data suggest that non-index patients have a considerably greater awareness of their clinical status, as expected. As a consequence, they were probably more alerted at the age of their very first epistaxis episodes (the disease onset for most of them) and were likely more accurate to record disease onset, since they were able to recognize it as the same stigmata affecting one of their ascendants/older siblings (usually representing the index case). The fact that the non-index cases receive a definite diagnosis in shorter time also depends to a second, synergistic factor, involving the physician’s ability to diagnose HHT more quickly due to the presence of a known HHT-affected index case in the familial history. As shown by our study, the attainment of HHT definite diagnosis in the first member of a family conveys a marked reduction in diagnostic delay of the index-case relatives, given that non-index cases receive a definite diagnosis 6.4 yrs on average after the index-case diagnosis, thereby representing a consistent improvement with respect to the time lag elapsing from their clinical onset (22.6 yrs). The relationship between index/non-index status and DTL proved to be statistically significant in both univariate and multivariate analysis, even though it failed to reach significance when age at interview was included in the multivariate analysis. This suggests that age acts as a confounding factor in the analysis, likely due to the expected relationship existing between index/non-index status and older age, being the non-index cases usually descendants or younger siblings than their index cases.

It is plausible that the presence of a known certainly HHT-affected relative in non-index’s familial history might help the diagnostician in at least three ways: by heighten knowledge of this disease in the referred physician, by providing a major criterion for clinical diagnosis (since the presence of a certainly affected 1^st^-degree relative is one of the four Curaçao criteria for clinical HHT diagnosis [14]), and by permitting genetic testing (in that genetic testing can be carried out only when the disease-causing mutation in either *ENG* or *ACVRL1* gene has been previously identified in an index-case with certain HHT [6]). The benefit of availability of genetic testing, very well evidenced by the difference in DTL among the non-index subgroup (14.4 years vs. 27 years in genetically-diagnosed vs. clinically-diagnosed, respectively) should prompt Institutions, physicians and Patients’ Associations to remove as many barriers as possible to the employment of genetic screening for early diagnosis, thus far an underused option [29]. The correlation of DTL with the mutated gene was far less clear, since univariate analysis found no significant difference in HHT1 vs. HHT2 patients, and multivariate analysis showed a correlation which, however, failed to reach statistical significance after removal of age from the model. A possible reason underlying the unclear role of mutated gene in DTL might consist in the prominent HHT2 composition of our cohort, as commonly observed in the Mediterranean countries [30], which might obscure the mutated-gene-contribution to the DTL.

Regarding the educational level of patients, our results demonstrated a gradual and significant decrease in DTL between people with different educational backgrounds. The relationship between educational level and DTL proved to be statistically significant in both univariate and multivariate analysis. The observation that education grade is one of the major factors affecting DTL can be explained by the well-known correlation between educational attainment and people’s behavior approach to their health status. Furthermore, subjects with higher education levels can usually benefit from increased social relationships and have an easier access to informative sources, such as the web-based tools, providing the necessary knowledge to reach the goal of a correct diagnosis.

Beside a high DTL, we also observed a long RTL. These considerations pinpoint the attention towards the insufficient knowledge of HHT among society. Owing to its extreme variability in manifestations, HHT not only suffers from poor recognition by physicians, but also by patients themselves. Since epistaxis can be present also in the general population, at times not related with a particular disease, nosebleeds may be discounted for many years as an annoyance within the range of normality, rather than perceived as the symptom of an underlying disease, by many patients/families [31]. This issue is currently being addressed by the HHT Foundation International, in order to improve awareness of this disease among as-yet undiagnosed HHT individuals [32].

Finally, some historical notes are worth of mention when discussing the long diagnostic delay in HHT, as well as in many other rare diseases. The time needed to accomplish a definite correct diagnosis depends on the complexity of essential diagnostic steps and the cognitive biases of the physician. A correct diagnosis will be formulated as quickly as the disease in question is retrieved from the physician’s working memory [33]. Cognitive biases, leading to misdiagnosis, might be synthesized by the statement “We recognize what we already know”, historically attributed to Socrates (V^th^ Century B.C.). HHT, or the Rendu-Osler-Weber disorder, is an example of this phenomenon. Another of such example is Cushing’s syndrome, often characterized by clinical heterogeneity and misdiagnosis [33,34]. Notably, the first typical description of a patient with Cushing’s syndrome was probably made by Sir William Osler, actually Harvey Cushing’s mentor [34] and one of the three eponyms of Rendu-Osler-Weber disorder. However, Osler was unable to properly acknowledge the existence of this new syndrome and formulated an incorrect diagnosis. Thus, Cushing’s syndrome was shed light onto only 20 years later, representing a typical instance of diagnostic delay, which some authors [34] have proposed to name “Osler’s phenomenon”.

## Conclusions

Patients with HHT experience a very long diagnostic delay before definitely attaining the correct diagnosis. Considerable endeavors are still required to spread awareness of this disease among health care professionals and patients’ relatives, in order to reduce the inacceptably long time lag associated with HHT diagnosis.

## Abbreviations

HHT, Hereditary Haemorrhagic Telangiectasia; AVMs, Arterio-Venous Malformations; RTL, Referral Time Lag; DTLD, Diagnosis Time Lag; LFID, Time Lag From Index Diagnosis; CAVMs, Cerebral Arterio-Venous Malformations; PAVMs, Pulmonary Arterio-Venous Malformations; HAVMs, Hepatic Arterio-Venous Malformations; BA, Brain Abscess; BS, Brain Stroke; TIA, Transient Ischemic Attack; ICH, Intracranial Haemorrhage; PRH, Pulmonary Haemorrhage; GRH, Haemorrhage from Large Gastrointestinal Colonic AVM; SZ, Seizures; HOF, High-Output Heart Failure; LINS, Liver Insufficiency; OLTx, Orthotopic Liver Transplantation.

## **Competing interests**

The authors declare that they have no competing interests.

## **Authors’ contributions**

PP participated in data collection and drafted the manuscript, GML participated in data analysis and drafted the manuscript; PS, PL and RV participated in data collection; MC and DS performed statistical analysis; VT and FR supervised the study process; GL, AS and NR edited the manuscript for important intellectual content; CS conceived the study and revised the final version of the manuscript. PP, GML, PS should be considered joint first Authors of this work. All authors read and approved the final manuscript.
